# Impaired synaptic plasticity and decreased excitability of hippocampal glutamatergic neurons mediated by BDNF downregulation contribute to cognitive dysfunction in mice induced by repeated neonatal exposure to ketamine

**DOI:** 10.1111/cns.14604

**Published:** 2024-02-08

**Authors:** Jie Wan, Linhui Ma, Xinhao Jiao, Wei Dong, Jiatao Lin, Yongkang Qiu, Weifeng Wu, Qiang Liu, Chen Chen, He Huang, Shuai Li, Hui Zheng, Yuqing Wu

**Affiliations:** ^1^ Jiangsu Province Key Laboratory of Anesthesiology, Jiangsu Province Key Laboratory of Anesthesia and Analgesia Application Technology, NMPA Key Laboratory for Research and Evaluation of Narcotic and Psychotropic Drugs Xuzhou Medical University Xuzhou China; ^2^ Department of Anesthesiology, National Cancer Center/National Clinical Research Center for Cancer/Cancer Hospital Chinese Academy of Medical Sciences and Peking Union Medical College Beijing China

**Keywords:** BDNF, cognitive dysfunction, excitability, glutamatergic neuron, ketamine, neonatal, synaptic plasticity

## Abstract

**Aim:**

Repeated exposure to ketamine during the neonatal period in mice leads to cognitive impairments in adulthood. These impairments are likely caused by synaptic plasticity and excitability damage. We investigated the precise role of brain‐derived neurotrophic factor (BDNF) in the cognitive impairments induced by repeated ketamine exposure during the neonatal period.

**Methods:**

We evaluated the cognitive function of mice using the Morris water maze test and novel object recognition test. Western blotting and immunofluorescence were used to detect the protein levels of BDNF. Western blotting, Golgi‐Cox staining, transmission electron microscopy, and long‐term potentiation (LTP) recordings were used to assess synaptic plasticity in the hippocampus. The excitability of neurons was evaluated using c‐Fos. In the intervention experiment, pAdeno‐CaMKIIα‐BDNF‐mNeuronGreen was injected into the hippocampal CA1 region of mice to increase the level of BDNF. The excitability of neurons was enhanced using a chemogenetic approach.

**Results:**

Our findings suggest that cognitive impairments in mice repeatedly exposed to ketamine during the neonatal period are associated with downregulated BDNF protein level, synaptic plasticity damage, and decreased excitability of glutamatergic neurons in the hippocampal CA1 region. Furthermore, the specific upregulation of BDNF in glutamatergic neurons of the hippocampal CA1 region and the enhancement of excitability can improve impaired synaptic plasticity and cognitive function in mice.

**Conclusion:**

BDNF downregulation mediates synaptic plasticity and excitability damage, leading to cognitive impairments in adulthood following repeated ketamine exposure during the neonatal period.

## Graphical abstract

This study illustrates the role of BDNF in cognitive dysfunction in adult mice that results from repeated ketamine exposure during development. After neonatal mice were anesthetized with ketamine multiple times, the level of BDNF in glutamatergic neurons in the CA1 area of the hippocampus was reduced, resulting in impaired neuronal synaptic plasticity and decreased neuronal excitability, ultimately leading to cognitive dysfunction in adulthood.

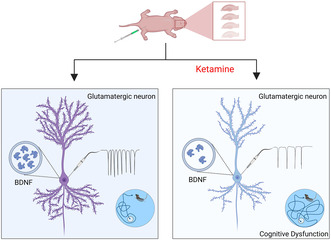

## 1 INTRODUCTION

General anesthesia during surgery is necessary for newborns, as it improves their comfort and cooperation during the procedure, promotes postoperative recovery, and leaves the child with no painful memories. In the United States alone, over 6 million children undergo anesthesia and surgery each year,^1^ with 14.9% of children receiving at least one general anesthesia before the age of three and 26% of children receiving repeated or prolonged anesthesia.^2^ Ketamine, a noncompetitive N‐methyl‐d‐aspartate receptor (NMDAR) antagonist, is widely used as an anesthetic for children due to its effective sedative and analgesic properties, as well as its cardiopulmonary stability effects.^3^ However, as an antagonist of NMDARs, ketamine can have adverse effects on neuronal plasticity. Structural plasticity can cause dendritic spine damage in prefrontal cortex neurons.^4^, ^5^ In terms of functional plasticity, it can affect neuronal excitability by inhibiting excitatory neurotransmission mediated by NMDA receptors.^6^ The newborn brain is still in the process of being fully developed, with dendrites and synapses undergoing rapid growth, and the neuronal functional network gradually maturing.^7^ Compared to the adult brain, it is more susceptible to the influence of external factors and drugs. In recent years, several large prospective and retrospective studies have suggested that exposure to anesthetic drugs during the neonatal period may affect pediatric brain development,^8^, ^9^, ^10^ with anesthesia before the age of four is an important risk factor for learning disabilities.^11^ Animal studies have also shown that exposure to anesthetic drugs during the neonatal period can lead to cognitive impairments in adulthood.^12^ Many preclinical studies have focused on neuroapoptosis and cognitive dysfunction following developmental anesthesia.^13^, ^14^ However, research on neuronal and synaptic plasticity and its underlying mechanisms is still incomplete, highlighting the need for further investigation into the toxicity of ketamine to the developing central nervous system. The neonatal period is characterized by the dynamic development of neuronal and synaptic plasticity, with the synaptic plasticity of hippocampal CA1 neurons being crucial for learning, memory, and cognitive function.^15^ Damage to synaptic plasticity significantly affects cognitive function.^16^ The transmission of signals between synapses relies on the involvement of numerous receptors and ion channels, which are essential for neuronal excitability.^17^ Alterations in the levels of major excitatory neurotransmitters and the glutamatergic system within the central nervous system can impact cognitive function.^18^ Neurotrophic factors are a family of secreted proteins that support neuronal growth, survival, and differentiation and have been extensively studied for decades due to their potent and diverse effects on neuronal physiology and therapeutic prospects. Brain‐derived neurotrophic factor (BDNF) is an important member of this family and plays a crucial role in the molecular mechanisms underlying synaptic plasticity.^19^ It is very important due to its pleiotropic effects within the central nervous system (CNS) and its role in various brain disorders. BDNF not only regulates the growth of dendrites during the developmental period^20^ but also modulates synaptic transmission^21^ and plays a critical role in the stability of synaptic plasticity.^22^ At presynaptic sites, BDNF enhances the exocytosis of glutamate‐containing synaptic vesicles, thereby further enhancing presynaptic neurotransmitter release.^23^, ^24^ Evidence suggests that BDNF is involved in inducing various forms of synaptic plasticity, such as long‐term potentiation (LTP),^25^ and plays a role in different stages of memory and cognitive function.^26^ Growing evidence from animal models and epidemiological studies has raised concerns about the long‐term or repeated use of general anesthesia in infants and young children. Therefore, in this study, early exposure to anesthetic drugs was simulated in mice by continuous anesthesia for 3 days starting at postnatal day 7 (PND‐7).^27^ The aim of this study was to determine the impact of BDNF on the synaptic plasticity, neuronal excitability, and cognitive function changes induced by repeated exposure to ketamine in newborns, further elucidating the mechanisms underlying the developmental brain neurotoxicity of ketamine and providing new insights and targets for implementing targeted neuroprotective strategies to improve the safety of anesthesia in children and infants.

## 2 MATERIALS AND METHODS

### 2.1 Animals

All animal experiments were approved by the Institutional Animal Care and Use Committee of Xuzhou Medical University (L20210113006). Pregnant C57BL/6J mice were produced by Xuzhou Medical University Experimental Animal Center. The Vglut1‐ires‐Cre (Vglut1‐Cre) mice used in the chemogenetic experiments were produced by Jackson Laboratory and were conducted by breeding homozygous mice to generate genotyped mice. Timed‐pregnant mice were housed at a temperature of 24 ± 1°C and humidity of 50 ± 10% with a 12‐h light/dark cycle and had free access to food and water. The animals were randomly assigned to different groups, and after each injection, they were returned to their respective rooms.

### 2.2 Neonatal anesthetized mouse model

The newborn mice were subjected to multiple anesthesia experiments based on previous research.^12^ PND‐7 mice (half male, half female, weighing 3–5 g) were selected. In the ketamine group, mice were intraperitoneally injected with 20 mg/kg ketamine for 3 consecutive days, with three injections administered each day at 1‐h intervals. The mice in the control group were also removed from mother care to receive an equivalent volume of normal saline injection three times a day at 1‐h intervals for three consecutive days. Subsequently, the mice were placed in an incubator with 60% oxygen for recovery and returned to their mother after complete recuperation.

### 2.3 Viral vectors and stereotaxic injection

The viral vectors pAdeno‐CaMKIIα‐mNeuronGreen (Ad‐mNeuronGreen) and pAdeno‐CaMKIIα‐BDNF‐mNeuronGreen (Ad‐BDNF), AAV9‐EF1α‐DIO‐mCherry (AAV‐mCherry), and AAV9‐EF1α‐DIO‐hM3Dq‐mCherry (AAV‐hM3Dq) were obtained from OBIO Technology (Shanghai) Corp., Ltd. and BrainVTA Corp., Ltd., respectively. Stereotaxic surgeries were carried out under general anesthesia where 3% sevoflurane was used for induction and hypothermic anesthesia for maintenance respectively. Hypothermic anesthesia was according to the protocol reported before,^28^ and mice were immersed in ice for 3 min but covered with filter paper to avoid the direct touch of skin to ice. The stereotaxic injection procedure was taken on a cool pad to keep mice immobile. Ad‐mNeuronGreen and Ad‐BDNF (0.1 μL on each side) were bilaterally microinjected into the hippocampal CA1 area (anterior–posterior, AP = 1.4 mm; medial‐lateral, ML = ±0.8 mm; dorsal–ventral, DV = −1.2 mm from the posterior fontanelle) at a rate of 20 nL/min in PND‐3 mice. AAV‐mCherry and AAV‐hM3Dq (0.2 μL on each side) were bilaterally microinjected into the hippocampal CA1 area (AP = −1.94 mm, ML = ±1.2 mm, DV = −1.5 mm from bregma) at a rate of 40 nL/min in PND‐40 mice. The mNeuronGreen or mCherry fluorescence signal in the hippocampal brain tissue sections confirmed the site of virus transfection. After the operation, mice were warmed gently in the warm cage to recover before transferring to the home cage.

Fluorescent images were captured using laser scanning confocal microscopy (Leica STELLARIS 5, Germany) to confirm the efficiency of virus expression.

### 2.4 Western blotting (WB)

The hippocampal tissue was homogenized using RIPA lysis buffer (Beyotime) containing PMSF. Protein concentrations were measured using a BCA kit (Beyotime) and adjusted with RIPA lysis buffer. After 5 min of boiling, the samples were stored at −20°C. Next, the proteins were separated using 10% gradient sodium dodecyl sulfate–polyacrylamide electrophoresis (SDS–PAGE) and transferred to PVDF membranes (Merck Millipore, ISEQ00010). The membranes were blocked with 5% nonfat milk for 2 h and then incubated overnight at 4°C with primary antibodies, including anti‐BDNF (1:3000, ab108319, Rabbit, Abcam), anti‐PSD95 (1:1000, 2507S, Rabbit, Cell Signaling Technology), and anti‐β‐actin (1:2000, AC004, Mouse, ABclonal, China). Subsequently, the membranes were incubated with horseradish peroxidase binding antibodies (1:2000, A0208, goat anti‐rabbit; A0216, goat anti‐mouse, Beyotime) for 2 h at room temperature. Protein bands on the membranes were visualized using ECL detection equipment (Beyotime) and quantified using ImageJ software.

### 2.5 Immunofluorescence

Mice were intracardially perfused with 0.9% saline followed by 4% paraformaldehyde after deep anesthesia by sevoflurane. Mouse brains were harvested and fixed in 4% paraformaldehyde for 6–8 h, and then the brains were subsequently dehydrated in 30% sucrose for 3 days. Using a freezing microtome (CM1950, Leica), 20‐μm‐thick coronal sections of the mouse brains were prepared. The brain sections were then blocked with 10% goat serum after the membranes were ruptured with 0.8% PBST. For immunofluorescence staining, the brain sections were incubated at 4°C with primary antibodies for 3 days and nights. The primary antibodies used were as follows: anti‐BDNF (1:100, ab108319, Rabbit, Abcam) mixed with anti‐GAD67 (1:300, ab26116, Mouse, Abcam); anti‐BDNF mixed with anti‐CamKIIα (1:300, 50049S, Mouse, Cell Signaling Technology); and anti‐c‐Fos (1:400, 2250, Rabbit, Cell Signaling Technology) mixed with anti‐GAD67 and anti‐c‐Fos mixed with anti‐CamKIIα. Afterward, the brain sections were probed with fluorescent secondary antibodies (1:400, ab150080, goat anti‐rabbit; ab150113, goat anti‐mouse; ab150115, goat anti‐mouse, Abcam) for 1 h at 37°C. Finally, DAPI (ab104139, Abcam) was used to stain the nucleus, and the staining outcomes were viewed using a confocal microscope (FV1000, Olympus).

### 2.6 Golgi‐Cox staining

To evaluate the dendritic development of neurons, Golgi‐Cox staining was performed using the Golgi‐Cox OptimStain PreKit (HiTO, USA) according to the manufacturer's instructions. Two sections from each rat brain were observed under a microscope, and one pyramidal neuron from the hippocampal CA1 region was analyzed from each section. For the analysis of dendritic spine density, one apical dendrite was selected from each pyramidal cell, and the dendritic spines along the full length of the branch were counted. The images were taken under a microscope (BX53, Olympus) at magnifications of 200× and 600× and analyzed using ImageJ software. Twelve neurons were counted for statistical analysis. The analysis was performed by an observer who was blinded to the treatment conditions. Sholl analysis was used to evaluate the number of dendritic branches of the neurons. Images of a single CA1 neuron were taken at 200× magnification under a microscope, and the target neuron was tracked and reshaped using ImageJ software. A circle was drawn around the cell body as the center, with the radius increasing by 20 μm each time. The number of intersections between the circles and dendrites was counted as the number of dendritic branches corresponding to the different radii. To evaluate the dendritic spine density, a field containing dendrites in the CA1 region was photographed under a microscope using a 600× oil immersion lens. Dendrites were uniformly selected from the straighter regions of the secondary dendrites. The dendritic spine density was measured as the number of dendritic spines in each 10 μm‐length of the branch.

### 2.7 Long‐term potentiation (LTP) recordings

After mice were perfused with frozen cutting solution preoxygenated with 95% oxygen and 5% carbon dioxide under sevoflurane anesthesia, the mouse brains were quickly extracted and the dorsal hippocampus was cut into coronal sections (300 μm thick) in cutting solution using a microtome (Leica VT 1200S). The brain sections were incubated in oxygenated artificial cerebrospinal fluid (ACSF) for 1 h at 32°C. Referring to previous literature,^29^ Schaffer collateral branches were stimulated with concentric stimulating electrodes, and the field excitatory postsynaptic potential (fEPSP) of the CA1 area was recorded using a glass electrode filled with ACSF (tip resistance 3–5 MΩ) which was pulled from borosilicate glass microtubule (Sutter Instrument; O.D.: 1.5 mm, I.D.: 0.86 mm). The activity was amplified by the microelectrode amplifier MultiClamper 700‐B (Axon Instruments) and then transferred into the analog‐to‐digital converter (Axon Instruments), acquired and analyzed by acquisition software Clampfit 7.0 (Axon Instruments). The fEPSP with a stable slope baseline was collected for 10 min under the basal stimulation delivered by a stimulus isolator (ISO‐Flex) whose intensity evoked 50% of the maximum fEPSP, and the frequency was 0.033 Hz. The 20%–80% slope (in mV/ms) of each fEPSP was quantified and 6 repetitive data were averaged. Three trains of high‐frequency stimulation (HFS; duration 1 s at 100 Hz with a 20‐s interval for each train) were delivered by Clampfit 7.0 and stimulus isolator to the slice to induce long‐term potentiation (LTP). After HFS, the changes in the fEPSP and its slope were monitored for an additional 1 h, and the average slopes during the last 10 min were analyzed.

### 2.8 Transmission electron microscopy

Harvested hippocampal tissue (1 × 1 × 1 mm^3^) was prefixed with a mixture of 2.5% glutaraldehyde and 4% paraformaldehyde for more than 24 h. Then, the brain tissue was fixed with 1% osmic acid for 2 h. After fixation, the brain tissue underwent gradient dehydration with ethanol and acetone before being submerged and implanted in Epon resin. Subsequently, an ultrathin microtome ((UC7rt) A‐1170) was used to cut the tissue into ultrathin sections (70 nm thick). These sections were then collected on copper mesh and stained with uranium acetate and lead citrate.^30^, ^31^ Photographs of the asymmetric synapses that mediate excitatory conduction were taken using a transmission electron microscope (Tecnai G2S pirit Twin). Twenty‐four synaptic structures from six mice per group were selected for analysis under a 5800× microscope. Using ImageJ software, the thickness of the postsynaptic density (PSD) and the synaptic cleft (SC) of a synapse was individually measured as the average length of the vertical lines from the postsynaptic membrane to the synaptic complex and from the presynaptic membrane to the postsynaptic membrane at five different locations. The measurement of the length and the chord of the active zone was also conducted by ImageJ, where the active zone length divided by the chord length resulted in the synaptic curvature. Finally, the average thickness of the PSD, SC, active zone length, and synaptic curvature across all synapses in an image was used as a single piece of data.

### 2.9 Chemogenetic manipulations

Based on previous experimental research,^32^ the chemical genetic virus was injected into the hippocampal CA1 region of mice for 3–4 weeks. Before behavioral tests, clozapine‐N‐oxide (CNO; Sigma–Aldrich, Saint Louis, MO, USA) was dissolved in dimethyl sulfoxide (DMSO), diluted with normal saline and intraperitoneally injected into mice at a dose of 3 mg/kg. When performing electrophysiological recording, the initial action potential firing of neurons was first recorded, 5 μM CNO was dripped into the incubation tank, and the action potential firing was recorded again after 5 min.

### 2.10 Behavioral tests

#### 2.10.1 Morris water maze test

Several groups of mice were reared to the age of 60 days. Hippocampus‐dependent spatial learning and memory abilities were tested using the Morris water maze (MWM) test, following the method reported in previous studies.^12^ The MWM test was conducted in a circular opaque pool (180 cm in diameter, 50 cm deep) filled with water to a depth of 30 cm, with the temperature maintained at 23 ± 1°C. An invisible platform (10 cm in diameter) was submerged 1 cm below the water surface and placed at the center of quadrant I, while the starting positions in quadrants I, II, III, and IV were equidistant from the pool's edge. The test was carried out over five consecutive days in a dark and quiet laboratory. Each mouse was trained four times per day, with randomized starting positions. The time taken for the mouse to find the platform (escape latency) was recorded, and if the mouse could not find the platform within 60 s, it was gently guided to the platform. The mouse was then allowed to stay on the platform for 10 s before the 60‐s latency to find the hidden platform was recorded. The average time of the four trials for each mouse represented its daily latency. On the sixth day, the hidden platform was removed, and the mouse was placed in the water in quadrant III, facing the pool wall. The mouse freely swam for 60 s, and the latency to first pass through the original platform location (escape latency), the number of times the mouse swam across the previous platform location, and the time the mouse stayed in the target quadrant within 60 s were recorded. The path of each mouse was tracked using a computerized video system. After each trial, the mouse was placed on a heating pad for 1–2 min until dry before being returned to its chamber. The data obtained from the MWM test were analyzed using MWM software.

#### 2.10.2 Novel object recognition test

The novel object recognition test (NORT) is an effective behavioral test commonly used to reveal the function of specific brain regions involved in memory. This test exploits the inherent preference of mice for novelty to assess their memory for previously encountered objects. The experiment was conducted in a cube measuring 40 cm in length, 40 cm in width, and 35 cm in height.^33^ Mice were given 10 min to acclimate to their surroundings. After a period of 1 h, the mice were exposed for 10 min to the cube, which contained two objects (object 1 and object 2). The amount of time they spent investigating each object was recorded. Following another hour, the mice were reintroduced to the cube for 5 min, but this time, object 2 was replaced with object 3. The time spent investigating both object 1 and object 3 was documented. The proportion of time spent exploring object 3 reflected a preference for novel stimuli.

### 2.11 Statistical analysis

The data were analyzed using GraphPad Prism 8.0 (GraphPad Software, Inc.) or SPSS (IBM). All data followed a normal distribution, which was verified by the Shapiro–Wilk test. The results are shown as the mean ± SEM. The unpaired *t*‐test was used to compare the differences between the two groups. To calculate the differences among the four groups in the BDNF overexpression experiment, two‐way ANOVA or one‐way ANOVA with Tukey's multiple comparison test was used. Two‐way repeated measures ANOVA was used to detect the differences in the escape latency considering time and group factors. For each experiment, the replicates (*n*) have been indicated in figure legends. Significance was determined by the corresponding statistical test listed in the figure legends. The threshold for significance was set at *p* < 0.05, *p < 0.05; **p < 0.01.

## 3 RESULTS

### 3.1 The protein level of BDNF in glutamatergic neurons in the hippocampal CA1 area of mice repeatedly exposed to ketamine during the neonatal period is downregulated

**FIGURE 1:**
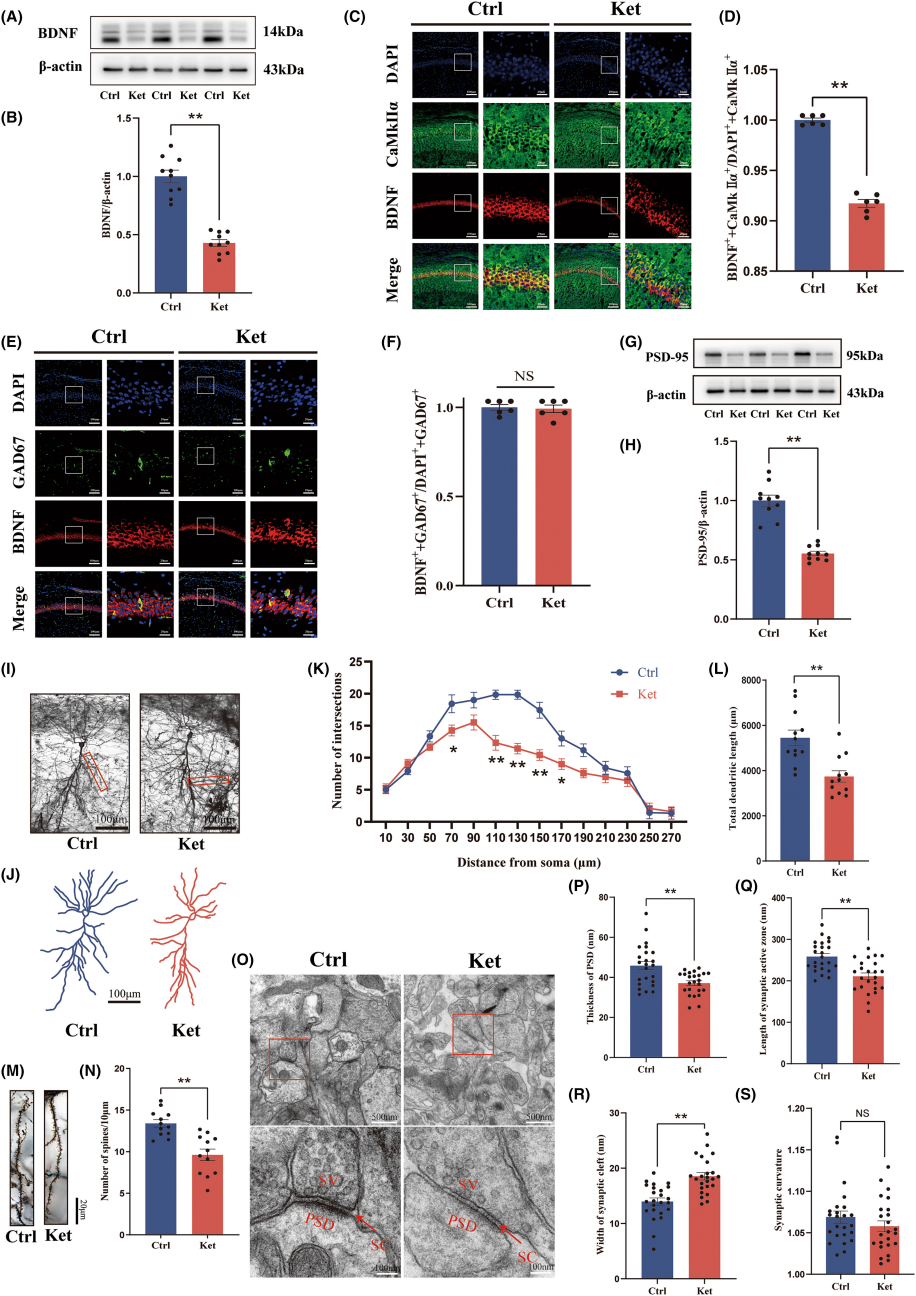
Repeated ketamine anesthesia in neonatal mice resulted in reduced BDNF protein levels in the glutamatergic neurons of the hippocampal CA1 area and impaired synaptic plasticity of neurons. (A) Representative western blot bands for BDNF. (B) Quantitative results showed that the protein level of BDNF in the hippocampus of mice in the Ket group was reduced. (C) Representative colabeling images of BDNF and CaMKIIα. (D) Quantitative results showed a decreased number of neurons co‐labeling of BDNF and CaMKIIα (BDNF‐positive glutamatergic neurons) in Ket group. (E) Representative colabeling images of BDNF and GAD67. (F) There was no significant difference in the colabeling of BDNF and GAD67 (BDNF‐positive GABAergic neurons) between the Ctrl and Ket groups. (G) Representative western blot bands for PSD‐95. (H) Quantitative results showed that the protein level of PSD‐95 in the hippocampus of mice in the Ket group was reduced. (I) A hippocampal profile image of Golgi staining of hippocampal CA1 neurons, 200× with camera tracings. Scare bar 100 μm. (J) Remodeling pictures of Golgi‐stained neurons. (K) Quantitation of dendritic intersections. (L) Quantitation of the total dendritic length. (M) A hippocampal profile image of Golgi staining of hippocampal CA1 neurons and 600× for spine counting. Scare bar 20 μm. (N) Quantitation of the dendritic spine density. (O) Synaptic structure of neurons in the hippocampal CA1 area under a transmission electron microscope. (P) Image analysis of PSD thickness. (Q) Image analysis of the length of the synaptic active zone. (R) Image analysis of the width of the synaptic cleft. (S) Image analysis of the synaptic curvature. Statistical analyses were *t*‐test (B, D, F, H, K, L, N, P–S) or multiple *t*‐test (K). *N* = 10 mice per group (B, H), *n* = 6 mice per group (D, F), *n* = 12 neurons from 6 mice per group (K, L, N), and *n* = 24 synapses from 6 mice per group (P–S). *p < 0.05; **p < 0.01; NS, no significance. Error bars indicate SEM. BDNF, brain‐derived neurotrophic factor; Ctrl, control; Ket, ketamine; PSD, postsynaptic density; SC, synaptic cleft; SV, synaptic vesicle.

During PND 7–9, newborn mice were intraperitoneally injected with either 20 mg/kg ketamine or physiological saline three times every hour. Twenty‐four hours after the last injection, the protein level of BDNF in the hippocampus of the Ctrl group and Ket group were examined using western blotting (WB) (Figure 1A,B). The results showed a significant decrease in BDNF protein level in the hippocampus of newborn mice after multiple administrations of ketamine at a dose of 20 mg/kg during the neonatal period.

Previously, studies have indicated the close relationship between the hippocampal CA1 region and learning and memory abilities.^34^, ^35^ We aimed to further clarify which type of neurons in the hippocampal CA1 region are affected by the decreased level of BDNF caused by ketamine. Since the neuron types in the hippocampal CA1 area mainly include glutamatergic neurons and GABAergic neurons, immunofluorescence staining was performed to co‐label the glutamatergic neuron‐specific marker CamkIIα with BDNF, as well as the GABAergic neuron‐specific marker GAD67 with BDNF (Figure 1C,E). The ratio of glutamatergic and GABAergic neurons which translate BDNF was calculated. The results showed a decreased number of neurons co‐labeled with BDNF and CamkIIα (BDNF^+^+ CamkIIα^+^) in the ketamine group compared to the Ctrl group (Figure 1D), while no significant changes were observed in the number of neurons co‐labeled with BDNF and GAD67 (BDNF^+^+GAD67^+^) (Figure 1F). These results indicate that the downregulation of BDNF after multiple exposures to ketamine anesthesia during the neonatal period mainly affects the glutamatergic neurons of the hippocampal CA1 region.

### 3.2 The synaptic plasticity and excitability of glutamatergic neurons in the hippocampal CA1 area of mice repeatedly exposed to ketamine during the neonatal period are reduced

The postsynaptic density protein‐95 (PSD95) is the most abundant scaffold protein of the dendritic spine which is located in the PSD of glutamatergic synapses. PSD95 is closely related to the organization of PSD structure through the interaction with other membrane receptors and ion channels, which is the foundation of structural and functional synaptic plasticity.^36^ We tested the protein level of PSD95 in the hippocampus of newborn rats. The results showed that PSD95 levels were significantly reduced in the ketamine group (Figure 1G,H).

The effects of ketamine anesthesia on synaptic structural plasticity during the neonatal period were evaluated using Golgi staining at the microscopic level (Figure 1I). Representative images of neuronal remodeling are shown in Figure 1J. Sholl analysis was conducted on neurons, and the results demonstrated a significant reduction in the number of dendritic branches at radii of 70, 110, 130, 150, and 170 in the CA1 region of the neonatal rat hippocampus exposed to ketamine anesthesia (Figure 1K: 70,170: *p* < 0.05; 110, 130, 150: *p* < 0.01). The total length of dendrites was also significantly decreased (Figure 1L). Furthermore, the dendritic spine density of neurons significantly decreased after ketamine exposure during the neonatal period (Figure 1M,N).

To further assess synaptic plasticity, the ultrastructure of synapses was observed using transmission electron microscopy. Representative images are shown in Figure 1O. The synaptic structure in neonatal rats exposed to ketamine anesthesia exhibited clear damage, characterized by thinning of the presynaptic dense material, shortening of the active zone length, and widening of the synaptic cleft (Figure 1P–R). These changes are detrimental to synaptic signal transmission. However, there were no significant changes in synaptic curvature (Figure 1S). These results indicate that the structural plasticity of neurons is immediately impaired in newborns after repeated exposure to ketamine anesthesia.

**FIGURE 2:**
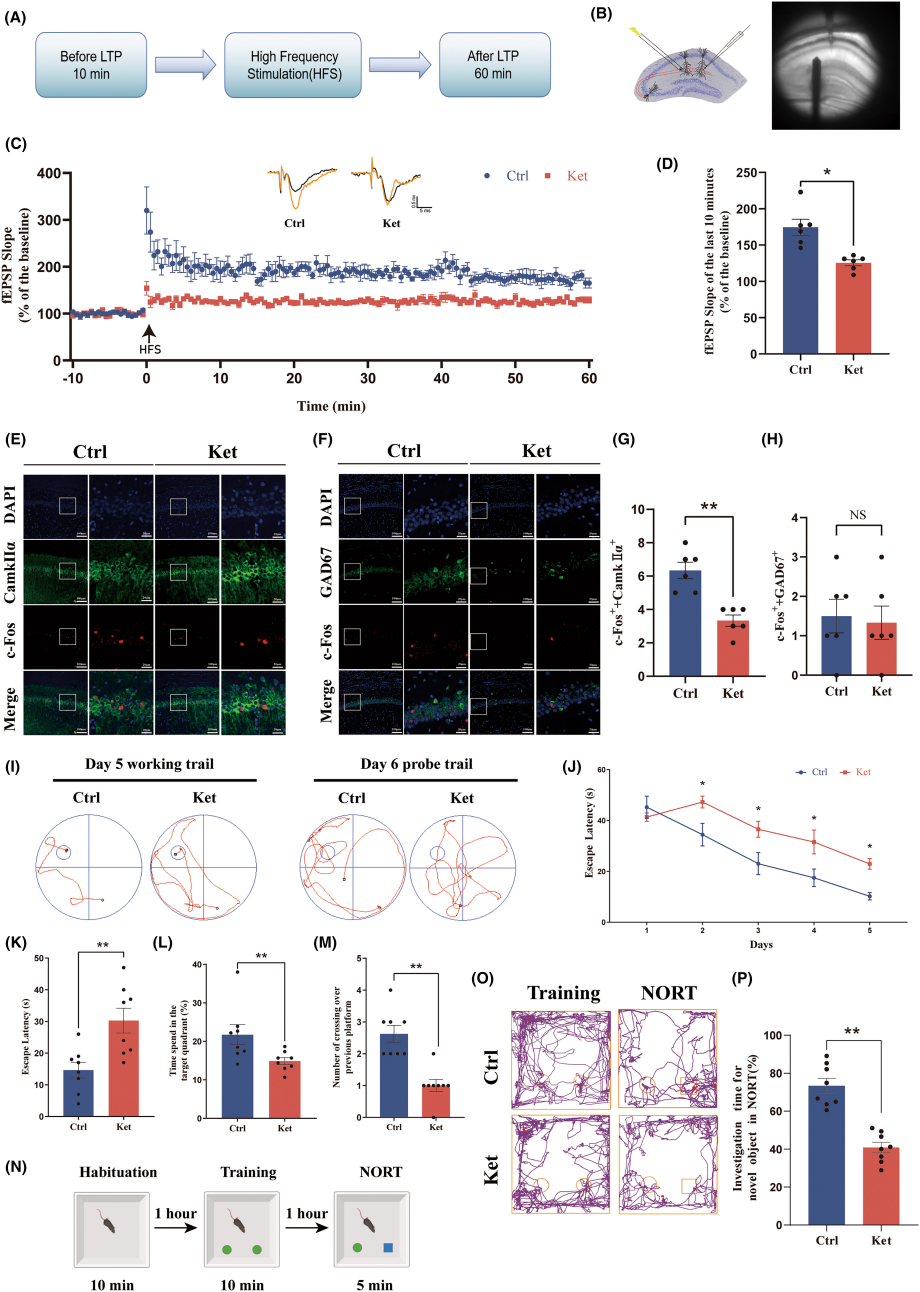
Repeated ketamine anesthesia in neonatal mice resulted in a reduction in LTP and the excitability of glutamatergic neurons and the development of cognitive dysfunction. (A) The experimental process of LTP. (B) Placement of stimulating and recording electrodes. (C) fEPSP slope before and after HFS was recorded as baseline (black, before HFS) and representative waveform (orange, after HFS). The arrow indicates the time point of the HFS application. (D) The average fEPSP slope at 50–60 min after HFS. (E) Representative colabeling images of c‐Fos and CaMKIIα. (F) Representative colabeling images of c‐Fos and GAD67. (G) The number of neurons that were c‐Fos+ and CaMKIIα+. (H) The number of neurons that were c‐Fos+ and GAD67+. (I) The typical paths of rats on the fifth training day and in the memory retrieval tests on the sixth day. (J) The latency to find the hidden platform during the training period from Day 1 to Day 5. (K) Escape latency in the memory retrieval tests on the sixth day. (L) The time spent in the target quadrant in the memory retrieval tests on the sixth day. (M) The number of times that the rats crossed the previous platform location within 60 s in the memory retrieval tests on the sixth day. (N) The experimental process of the NORT. (O) Representative tracks of the two groups in the NROT. (P) The percentage of investigation time for novel object exploration in the NORT. Statistical analyses were *t*‐test (D, G, H, K–M, P) or two‐way ANOVA (J). *N* = 6 mice per group (C, D, G, H), *n* = 8 mice per group (J–M, P). *p < 0.05; **p < 0.01; NS, no significance. Error bars indicate SEM. fEPSP, field excitatory postsynaptic potentials; HFS, high‐frequency stimulation; LTP, long‐term potentiation; NORT, novel object recognition test.

To evaluate long‐term potentiation (LTP) and synaptic transmission function, field excitatory postsynaptic potentials (fEPSPs) were recorded from CA3 to CA1 Schaffer collateral afferents following behavioral testing in mouse hippocampal CA1 slices (Figure 2A,B). Representative fEPSPs obtained before (blue) and after (red) high‐frequency stimulation (HFS) are shown in Figure 2C. Both normal mice and neonatal rats exposed to ketamine anesthesia exhibited stable LTP induced by HFS, but the magnitude of CA3–CA1 LTP was significantly reduced in the Ket group compared to the Ctrl group (Figure 2C). The average amplitude of fEPSPs within 50–60 min after stimulation significantly decreased from 174.5% ± 11.01% (Ctrl group) to 125.5% ± 4.105% (Ket group) (Figure 2D).

Following behavioral testing, c‐Fos was used to evaluate neuronal excitability, and CamkIIα and GAD67 were used to label glutamatergic and GABAergic neurons, respectively. The number of double‐stained neurons was counted using immunofluorescence staining to assess the activation of glutamatergic and GABAergic neurons in the CA1 region (Figure 2E,F). The number of activated glutamatergic neurons in the CA1 region was decreased in the ketamine group compared to the control group (Figure 2G), while there was no significant difference in the activation of GABAergic neurons (Figure 2H). These results indicate that ketamine exposure during the neonatal period leads to a decrease in glutamatergic neuron excitability in the CA1 region of the hippocampus, rather than GABAergic neurons.

### 3.3 Mice repeatedly exposed to ketamine during the neonatal period suffer cognitive impairment in adulthood

The influence of repeated ketamine anesthesia in early life on spatial learning and memory abilities in adulthood was assessed using the Morris water maze (MWM) test. The typical paths of mice on the fifth training day and the sixth testing day are shown in Figure 2I. As the training progressed, both groups of mice exhibited a decreasing trend in the latency to find the hidden platform, indicating a learning ability over time. However, starting from the second day, the ketamine group took a significantly longer time to find the hidden platform than the control group (Figure 2J). In the memory retrieval test on the sixth day, the ketamine group had significantly longer escape latencies than the control group (Figure 2K). Additionally, compared to the control group, the ketamine group had a significantly reduced time spent in the quadrant where the platform was located (Figure 2L). Moreover, the number of passes through the previous platform location was also significantly reduced in the ketamine group (Figure 2M).

Furthermore, the novel object recognition test (NORT) was also conducted to assess short‐term memory abilities in mice (Figure 2N). The typical paths of mice during training and testing are shown in Figure 2O. Interaction with objects in the training phase showed no significant difference between the two groups (Figure S1). The results showed that adult mice with multiple exposures to ketamine in early life had significantly reduced exploration time for the novel objects (Figure 2P). All the behavioral data indicate that multiple exposures to ketamine in early life impair learning and memory functions in adulthood.

### 3.4 Overexpression of BDNF in glutamatergic neurons in the hippocampal CA1 region prevents synaptic plasticity impairment and decreased excitability induced by multiple ketamine exposures during the neonatal period

**FIGURE 3:**
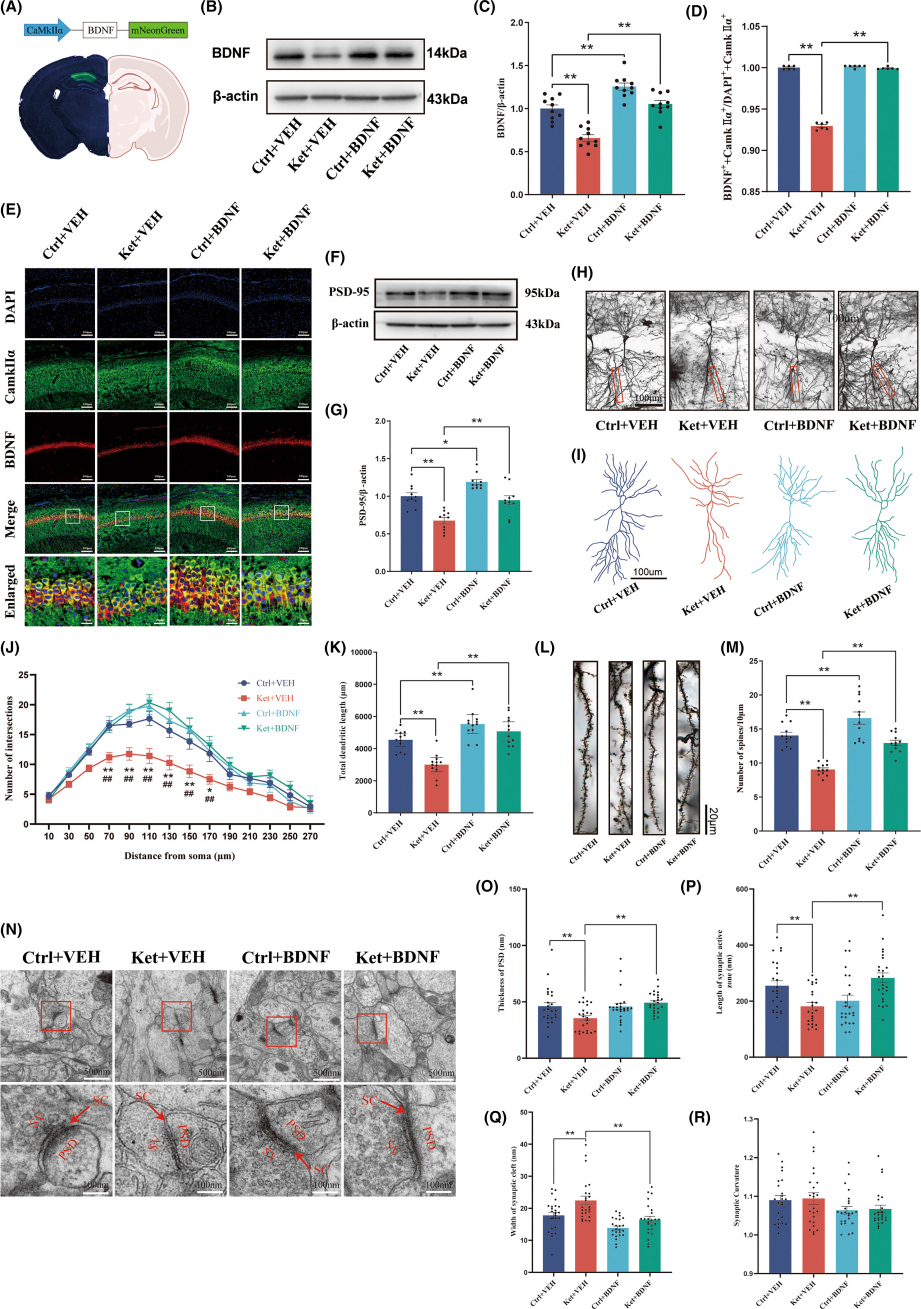
Overexpression of BDNF in glutamatergic neurons in the CA1 area of the hippocampus prevents the plasticity impairment of synaptic structures caused by multiple exposures to ketamine during the neonatal period. (A) Fluorescence images showing efficient expression of pAdeno‐CaMKIIα‐mNeuronGreen in the CA1 region. (B) Representative western blot bands for BDNF. (C) Quantification of BDNF level. (D) Quantitation of the ratio of BDNF^+^/CamkIIα^+^ neurons to DAPI^+^/CamkIIα^+^ neurons (the ratio of BDNF‐positive glutamatergic neurons to the glutamatergic neurons). (E) Representative colabeling images of BDNF and CaMKIIα. (F) Representative western blot bands for PSD‐95. (G) Quantification of PSD‐95 level. (H) A hippocampal profile image of Golgi staining of hippocampal CA1 neurons, 200× with camera tracings. (I) Remodeling pictures of Golgi‐stained neurons. (J) Quantitation of dendritic intersections. *p < 0.05; **p < 0.01 versus the Ctrl+VEH group; ^##^ *p* < 0.01 versus the Ket+BDNF group. (K) Quantitation of the total dendritic length. (L) A hippocampal profile image of Golgi staining of hippocampal CA1 neurons and 600× for spine counting. (M) Quantitation of the dendritic spine density. (N) Synaptic structure of neurons in the hippocampal CA1 area under a transmission electron microscope. (O) Image analysis of PSD thickness. (P) Image analysis of the length of the synaptic active zone. (Q) Image analysis of the synaptic cleft width. (R) Image analysis of the synaptic curvature. Statistical analyses were two‐way ANOVA (D, K, O, P) or one‐way ANOVA with Tukey's multiple comparisons test (C, G, J, M, Q, R). *N* = 10 mice per group (C, G), *n* = 6 mice per group (D), *n* = 12 neurons from 6 mice per group (J, K, M), *n* = 24 synapses from 6 mice per group (O–R). *p < 0.05; **p < 0.01; NS, no significance. Error bars indicate SEM. BDNF, brain‐derived neurotrophic factor; PSD, postsynaptic density; SC, synaptic cleft; SV, synaptic vesicle.

To achieve specific overexpression of BDNF in glutamatergic neurons in the CA1 region of the hippocampus, pAdeno‐CaMKIIα‐BDNF‐mNeuronGreen and pAdeno‐CamkIIα‐mNeonGreen (as control) were injected into the CA1 region of PND‐3 mice with a stereotaxic injection system (Figure 3A). Following ketamine anesthesia at PND7‐9, western blot analysis was performed at PND‐10, which showed that the protein level of BDNF was higher in the Ctrl+BDNF group than in the Ctrl+VEH group, while the level of BDNF in the Ket+VEH group remained lower than that in the Ctrl+VEH group. However, the overexpression of BDNF in the Ket+BDNF group rescued the decrease in BDNF levels caused by ketamine (Figure 3B,C).

Immunofluorescence double staining for CamkIIα and BDNF was also performed at the same time points (Figure 3E). The level of BDNF in glutamatergic neurons in the Ket+VEH group was still lower than that in the Ctrl+VEH group. After overexpressing BDNF, there was no change in the number of CamkIIα and BDNF colabeled neurons in the Ctrl+BDNF group compared to the NS+VEH group, which was expected, as almost all glutamatergic neurons contain BDNF in normal mice. However, mice that received ketamine anesthesia during the neonatal period and injected with the virus exhibited restored BDNF levels in glutamatergic neurons (Figure 3D).

Neuronal structural synaptic plasticity was evaluated using western blotting, Golgi‐Cox staining, and transmission electron microscopy. Protein immunoblotting results showed that the protein level of PSD‐95 was significantly downregulated in the Ket+VEH group compared to the control group, but upregulated in the Ket+BDNF group compared to the Ket+VEH group and showed no significant difference compared to the control group (Figure 3F,G).

Representative images and reconstructions of CA1 pyramidal neurons in each group are shown in Figure 3H,I. Sholl analysis was performed to evaluate dendritic branching in each group. At distances of 70, 90, 110, 130, 150, and 170 μm from the soma, there was a significant reduction in the number of dendritic branches in the Ket+VEH group compared to the control group. However, in the Ket+BDNF group, there was a significant increase in the number of branches at these radii, which were able to recover to normal levels (Figure 3J). Total dendritic length and dendritic spine density were measured, and it was found that overexpression of BDNF in glutamatergic neurons in the CA1 region caused an increase in dendritic length and spine density. Compared to the Ket+VEH group, the Ket+BDNF group showed a significant increase in dendritic length and spine density, reaching normal levels (Figure 3K–M).

Transmission electron microscopy was used to observe the ultrastructure of synapses (Figure 3N). Compared to the Ket+VEH group, the Ket+BDNF group showed a significant increase in the thickness of the postsynaptic density, length of the active zone, and narrowing of the synaptic cleft (Figure 3O–Q). There was no significant difference in curvature between the groups (Figure 3R), indicating that BDNF can counteract the synaptic ultrastructural damage caused by ketamine.

**FIGURE 4:**
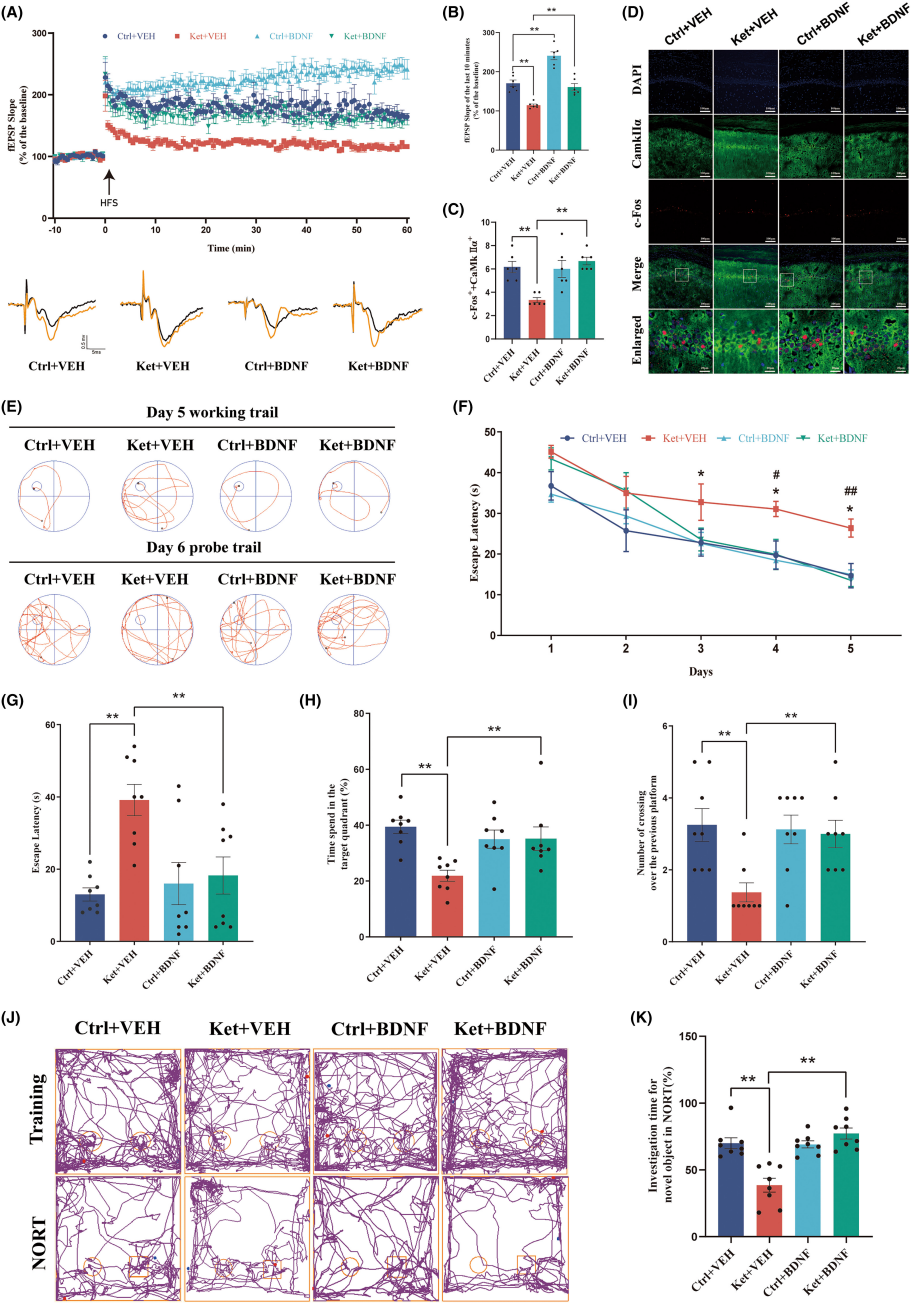
Overexpression of BDNF in glutamatergic neurons in the hippocampal CA1 area prevents the attenuation of LTP, reduced neuronal excitability, and cognitive dysfunction caused by multiple exposures to ketamine during the neonatal period. (A) fEPSP slope before and after HFS was recorded as baseline (black, before HFS) and representative waveform (orange, after HFS). The arrow indicates the time point of the HFS application. (B) The average fEPSP slope at 50–60 min after HFS. (C) The number of neurons that were c‐Fos+ and CaMKIIα+. (D) Representative colabeling images of c‐Fos and CaMKIIα. (E) The typical paths of rats on the fifth training day and in the memory retrieval tests on the sixth day. (F) The latency to find the hidden platform during the training period from day 1 to day 5. *p < 0.05 versus the Ctrl+VEH group; ^#^ *p* < 0.05; ^##^ *p* < 0.01 versus the Ket+BDNF group. (G) Escape latency in the memory retrieval tests on the sixth day. (H) The time spent in the target quadrant in the memory retrieval tests on the sixth day. (I) The number of times that the rats crossed the previous platform location within 60 s in the memory retrieval tests on the sixth day. (J) Representative tracks of the four groups in the NROT. (K) The percentage of investigation time for novel object exploration in the NORT. Statistical analyses were two‐way ANOVA (C, F, G–I, K) or one‐way ANOVA with Tukey's multiple comparisons test (B). *N* = 6 mice per group (A–C), *n* = 8 mice per group (F–I, K). *p < 0.05; **p < 0.01. Error bars indicate SEM. fEPSP, field excitatory postsynaptic potentials; HFS, high‐frequency stimulation; LTP, long‐term potentiation; NORT, novel object recognition test.

These results demonstrate that multiple exposures to ketamine during the neonatal period can lead to synaptic plasticity impairment in hippocampal CA1 neurons. However, pre‐overexpression of BDNF in glutamatergic neurons can protect against this injury caused by ketamine. To evaluate synaptic plasticity, long‐term potentiation (LTP) was measured in the hippocampal CA3‐CA1 region of mice in each group (Figure 4A). Representative fEPSPs from each group are shown at the bottom of the graph (Figure 4A). Overexpression of BDNF significantly enhanced fEPSPs, and compared to the Ket+VEH group, the Ket+BDNF group showed a significant increase in the slope during the last 10 min (Figure 4B). This finding indicates that overexpression of BDNF in glutamatergic neurons in the CA1 region can effectively improve the impairment of LTP and synaptic plasticity caused by neonatal ketamine anesthesia.

Immunofluorescence double staining (Figure 4D) showed that the number of c‐Fos‐positive neurons in the Ket+BDNF group was higher than that in the Ket+VEH group, Ctrl+VEH group, Ctrl+BDNF group, and Ket+BDNF group, although there was no significant difference in the number of double‐positive neurons among these groups (Figure 4C). These results indicate that the decrease in excitability of glutamatergic neurons caused by multiple exposures to ketamine during the neonatal period was prevented by BDNF overexpression.

### 3.5 Overexpression of BDNF in glutamatergic neurons in hippocampal CA1 ameliorates cognitive impairment in adulthood induced by multiple neonatal ketamine exposures

During the Morris water maze experiment on postnatal day 60 (PND‐60), the typical movement trajectory of the last day of training is shown at the top of Figure 4E, while the typical movement trajectory of the exploration experiment on the sixth day is shown at the bottom of Figure 4E. Over the course of 5 consecutive days of training, the escape latency of all four groups decreased, indicating a learning ability in mice over time. There was no significant difference in escape latency between the Ctrl+VEH and Ctrl+BDNF groups. However, starting from the third training day, the Ket+VEH group showed a significantly longer escape latency than the Ctrl+VEH and Ket+BDNF groups (Figure 4F). In the spatial exploration experiment on the sixth day, the Ket+BDNF group had a much shorter escape latency than the Ket+VEH group (Figure 4G). The percentage of time spent in the quadrant where the platform was located and the time spent crossing the platform was significantly improved in the Ket+BDNF group (Figure 4H,I).

The novel object recognition test (NORT) was conducted on the second day after the Morris water maze. Interaction with objects in the training phase showed no significant difference among the four groups (Figure S1). The figure shows representative trajectories of mice from each group during training and testing (Figure 4J). The Ket+VEH group spent significantly less time exploring the novel object than the Ctrl+VEH and Ket+BDNF groups (Figure 4K).

These findings collectively indicate that overexpression of BDNF in glutamatergic neurons in the CA1 region of the hippocampus can prevent learning and memory impairments caused by multiple ketamine anesthesia exposures during the neonatal period.

### 3.6 Chemogenetic manipulation activates glutamatergic neurons in the hippocampal CA1 area and improves adult cognitive dysfunction induced by multiple exposures to ketamine during the neonatal period

**FIGURE 5:**
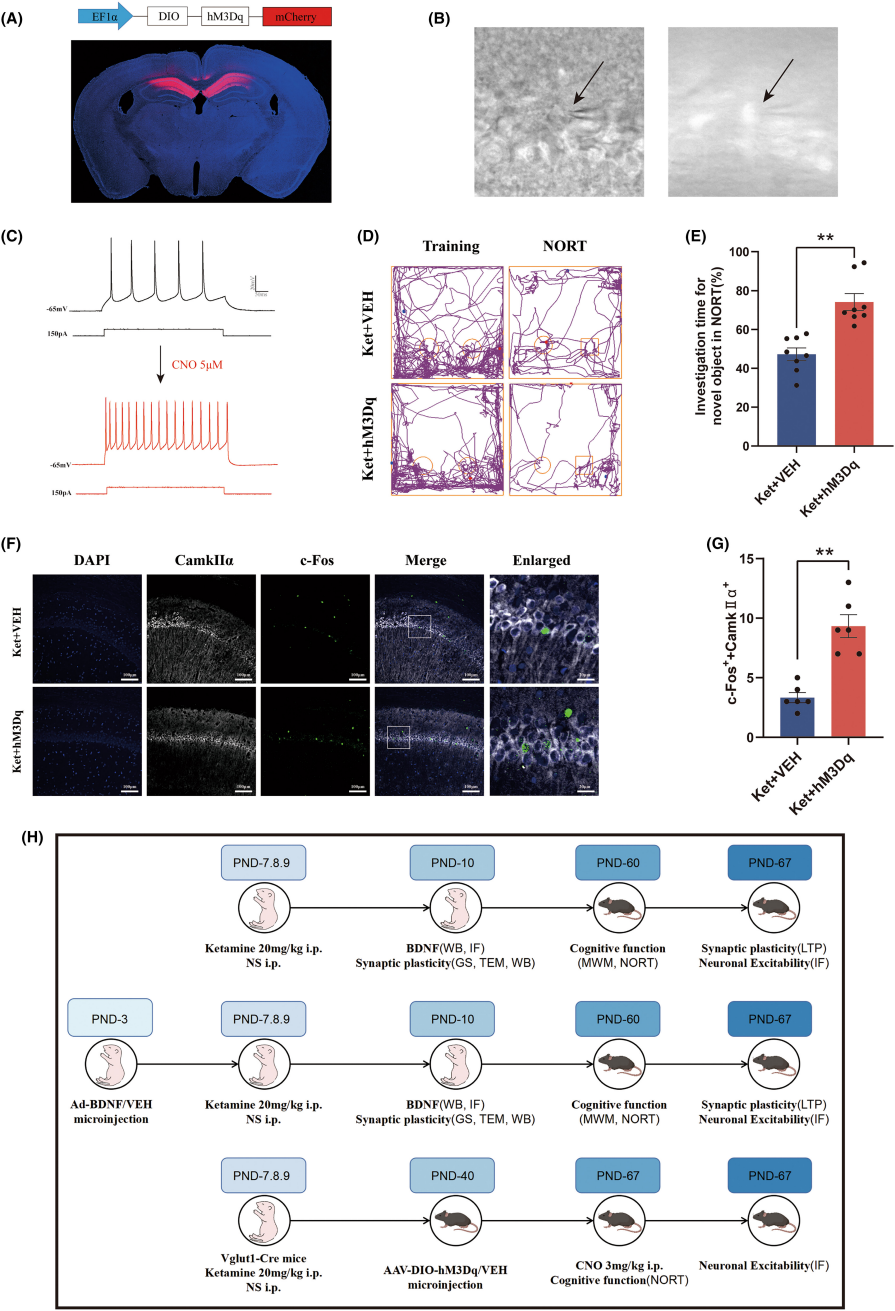
Activation of glutamatergic neurons in the hippocampal CA1 region can restore cognitive dysfunction in adulthood caused by multiple exposures to ketamine in neonates. (A) Fluorescence images showing efficient expression of AAV‐EF1α‐DIO‐hM3Dq‐mCherry in the CA1 region. (B) Whole‐cell patch‐clamp recordings of hm3Dq‐transfected neurons. (C) Bath application of CNO (5 μM) increased the firing rate of hM3Dq‐positive glutamatergic neurons injected with 150 pA current in vitro. (D) Representative tracks of the two groups in the NROT. (E) The percentage of investigation time for novel object exploration in the NORT. (F) Representative colabeling images of c‐Fos and CaMKIIα. (G) The number of neurons that were c‐Fos+ and CaMKIIα+. (H) Flow chart of the entire experiment. Statistical analyses were *t*‐test. *N* = 8 mice per group (E, G). **p < 0.01. Error bars indicate SEM. CNO, clozapine‐N‐oxide; LTP, long‐term potentiation; NORT, novel object recognition test.

The above results indicate that repeated ketamine anesthesia during the neonatal period leads to a decrease in the excitability of glutamatergic neurons. To further confirm the role of altered excitability of glutamatergic neurons in the hippocampal CA1 region in the learning and memory deficits of mice with ketamine‐induced injury, we used chemogenetic techniques to selectively activate glutamatergic neurons in the hippocampal CA1 region. First, on PND‐40, AAV9‐EF1α‐DIO‐hM3Dq‐mCherry was injected into the CA1 region of Vglut1‐Cre transgenic mice, allowing selective expression of hM3Dq in glutamatergic neurons, while AAV9‐EF1α‐DIO‐mCherry was injected into the control group. The injection sites are shown in Figure 5A. The effectiveness of CNO in activating hM3Dq‐expressing neurons was confirmed using a whole‐cell patch‐clamp technique, and Figure 5B shows live brain slices with mCherry fluorescence in CA1 neurons. Representative recordings showing evoked action potentials (150 pA) in neurons with mCherry fluorescence from mice expressing hM3Dq before and after administration of CNO (5 μM) (Figure 5C). The results show that CNO activates hM3Dq receptors and increases the action potential firing frequency of glutamatergic neurons in the CA1 area.

To evaluate the impact of the excitability of glutamatergic neurons in the hippocampal CA1 region on learning and memory, a novel object recognition test (NORT) was conducted in both groups of mice. Thirty minutes before the start of the experiment, a single intraperitoneal injection of CNO at a dose of 3 mg/kg was administered. Interaction with objects in the training phase showed no significant difference between the two groups (Figure S1). Figure 5D shows representative trajectories of the mice during the training and testing phases. After specific activation of glutamatergic neurons in the CA1 region with CNO, the ket+hM3Dq group mice showed a significant increase in exploratory time for the novel object compared to the Ket+mCherry group (Figure 5E), indicating better learning and memory abilities.

After the completion of the behavioral experiments, double immunofluorescence staining was performed on the activated neurons in the glutamatergic neurons (Figure 5F). The number of CaMKIIα and c‐Fos double‐positive cells in the hippocampal CA1 region was significantly increased in the Ket+hM3Dq group compared to the Ket+mCherry group (Figure 5G).

These results demonstrate that the chemogenetic techniques used in this study can effectively modulate the excitability of glutamatergic neurons in the hippocampal CA1 region. Activation of glutamatergic neurons in the CA1 region can significantly improve the cognitive impairments caused by early‐life exposure to ketamine. This finding further supports the notion that cognitive dysfunction in adulthood induced by neonatal ketamine anesthesia is likely achieved through a reduction in the excitability of glutamatergic neurons in the hippocampal CA1 region and that activation of these neurons can rescue cognitive deficits.

## 4 DISCUSSION

Many previous studies have shown the potential harm of general anesthetics to the developing brain and long‐term behavioral and cognitive development,^37^, ^38^ but with the not fully understood mechanism. The frequency and dose of anesthetic exposure during the neonatal period are closely related to the severity of neurotoxicity,^39^ and neonates with single‐dose anesthetic exposure showed unobvious effects in clinical trials.^9^ So based on previous research, we chose to inject clinical doses of ketamine (20 mg/kg) into newborn mice on the 7th, 8th, and 9th days after birth.^40^, ^41^, ^42^ Ketamine is a noncompetitive blocker of NMDA receptors (NMDARs).^43^ It has an analgesic effect without causing adverse reactions such as cardiovascular and respiratory depression that common anesthetics have. It is one of the general anesthetics commonly used in clinical anesthesia for children and infants.^44^ However, many studies are showing that repeated exposure to ketamine(clinical anesthetic doses) can cause cognitive dysfunction,^45^ which may be due to several processes including neuronal apoptosis,^46^ oxidative stress and neuroinflammation,^47^, ^48^ synaptic dysfunction,^49^, ^50^ and abnormal proliferation and differentiation of neural stem cells.^51^, ^52^ In this study, we explored the mechanism of cognitive dysfunction induced by neonatal ketamine exposure from the synaptic plasticity and neuronal excitability disorder perspective. In China, Esketamine, the enantiomer of ketamine has also been widely used in recent years. Some researchers have investigated the safety of supplemental use of Esketamine in cesarean delivery for pregnancy and neonates but focused on its short‐term effects.^53^, ^54^ It is also of great importance to compare the long‐term effects of these two enantiomers in our model in the future.

The brain growth spurt (BGS) is a critical period for synaptic development. The presence of many adverse stimuli during the BGS period can damage synaptic plasticity,^55^, ^56^, ^57^ which may lead to long‐term cognitive dysfunction in adolescence and adulthood. The hippocampus is a brain structure critical for cognitive function,^58^ the CA1 area of which has been proven to be closely related to cognitive function.^59^ Therefore, we focused on the CA1 area of the hippocampus and investigated the synaptic plasticity and neuronal excitability here following the experiment design illustrated in Figure 5H.

There are many different types of neurons in the brain, each with their specific functions,^60^ and glutamatergic neurons combined with GABAergic neurons constitute the neuronal microcircuits in the hippocampus.^61^ The brain‐derived neurotrophic factor BDNF is a member of the neurotrophic factor family. It is widely present in the central nervous system and plays a variety of roles.^62^ It can be produced and secreted by glutamatergic neurons and also exists in GABAergic interneurons.^63^ Many studies have shown that BDNF is closely related to the synaptic plasticity of neurons and enhances LTP,^64^ which was also confirmed in our research. Overexpression of BDNF can not only enhance neuronal synaptic plasticity and LTP but also the neuronal excitability of both glutamatergic and GABAergic neurons^65^, ^66^ and vice versa.^67^ In our study, we found that the protein level of BDNF in mice who received ketamine anesthesia multiple times during the neonatal period decreased. We also detected that this decrease mainly occurred in glutamatergic neurons by using immunofluorescence (Figure 1C). Since a recent study found that antidepressant doses of ketamine help maintain the glutamate‐excitatory/GABA‐inhibitory balance,^68^ we asked whether the much higher than antidepressant doses (clinical anesthetic doses) ketamine exposure could disrupt this balance which can be explained by the unbalanced decrease of BDNF. We found that the excitability of glutamatergic neurons was decreased in adulthood while GABAergic neurons were not significantly affected (Figure 2E,F), indicating that clinical doses of ketamine damaged the excitability of glutamatergic neurons and destroyed the local glutamate‐excitatory/GABA‐inhibitory balance in hippocampal CA1 region. This imbalance is also BDNF‐dependent, since it is successfully rescued by specific overexpression of BDNF in glutamatergic neurons. The unaffected GABAergic neurons' excitability after ketamine detected by the invariance of c‐fos‐positive GABAergic neurons is interesting but could be analyzed from several aspects. On the one hand, principal neurons in CA1 receive modulations of local interneurons but also projections from other distal regions, and inputs of these inhibitory interneurons are from not only the adjacent neurons but also cells in distal regions, excitatory or inhibitory,^69^, ^70^ which means the declined excitability of glutamatergic neurons does not necessarily lead to the downregulation of GABAergic neurons, and vice versa. These synaptic connections are complex and not totally clear now, and even the local microcircuit in the CA1 region has many types of interaction modules.^71^ In this study, we just focused on the CA1 region, while the changes of inputs from distal regions or the signal integration were not detected, so the decreased excitability of glutamatergic neurons could be the primary effect of ketamine exposure or secondary changes to other projections, and the unchanged c‐fos positive GABAergic neurons could also be the manifestation of integration of local and distal inputs. We wondered whether this could help explain the unchanged ratio of c‐fos positive interneurons in the CA1 region. On the other hand, the unchanged excitability of GABAergic after neonatal ketamine exposure does not imply that interneurons are immune to this neurotoxic effect. It has been reported that repeated neonatal ketamine exposures induce interneuron loss in the hippocampus.^72^ The changes in cell amount and associated compensatory modulations should also be taken into consideration in future studies.

There are also some shortcomings in this study. First, the excitability of neurons was investigated in our study, but these tests were somewhat limited and did not detect the dynamic process of real‐time excitability of neurons. In future studies, we will use in vivo Ca^+^ imaging, electrophysiology, and other techniques to more comprehensively investigate the excitability of neurons. Second, as the ligand of TrkB, BDNF takes part in its various roles by upregulating the phosphorylation of TrkB, which is constitutively expressed in many kinds of cells in the CNS. BDNF itself could be secreted by not only neurons but also glial cells, which hints at the cellular crosstalk behind the BDNF/TrkB signal.^73^ It has been reported that microglia regulate neuronal synaptic formation and plasticity through BDNF secretion^74^, ^75^ and BDNF could also bind with TrkB in microglia to induce neuroinflammation.^76^ Although the cellular interaction through BDNF/TrkB signal has been widely explored, seldom research was under the background of neonatal ketamine exposure. In our study, we investigated the level of BDNF in neurons and only focused on its role in synaptic plasticity regulation. Since neuroinflammation is also an important and unignorable factor affecting synaptic plasticity in this neurotoxicity process according to past studies,^47^, ^48^ it is of great importance to explore the potential role of BDNF‐mediated or microglia‐participated neuroinflammation mechanism further in the future. What is clear is that BDNF may be a potential target for the development of protective drugs against developmental neurotoxicity. Clinical translation of it should also consider the brain–blood barrier, and engineered BDNF‐producing cells may be a potential transport route.^77^ Apart from this, the agonist of TrkB could also upregulate the BDNF/TrkB signal but not be specific to certain types of neurons. However, several studies have reported the advantages of TrkB agonist for clinical translation because of its long half‐life and high efficiency which is superior to exogenous BDNF.^78^ So, further exploration of the rescue effects of TrkB agonist in this model is indeed crucial and meaningful in the future, and the neuron‐specific modulation of this agonist could even bring us a new method to realize the clinical translation. At the same time, the search for protective drugs can also focus on enhancing glutamatergic neuron excitability and restoring the glutamate‐excitatory/GABA‐inhibitory balance. Moreover, the brain is a very complex organization with many connections between various brain regions and various types of neurons. Although our study found that multiple exposures to ketamine during the neonatal period can affect the excitability of glutamatergic neurons in the microcircuit of the hippocampal CA1 region, whether this change in excitability caused changes in upstream neurons or downstream neurons and the relationships between neurons and glial cells are also of interest which will be elucidated in future studies.

## 5 CONCLUSION

**FIGURE 6:**
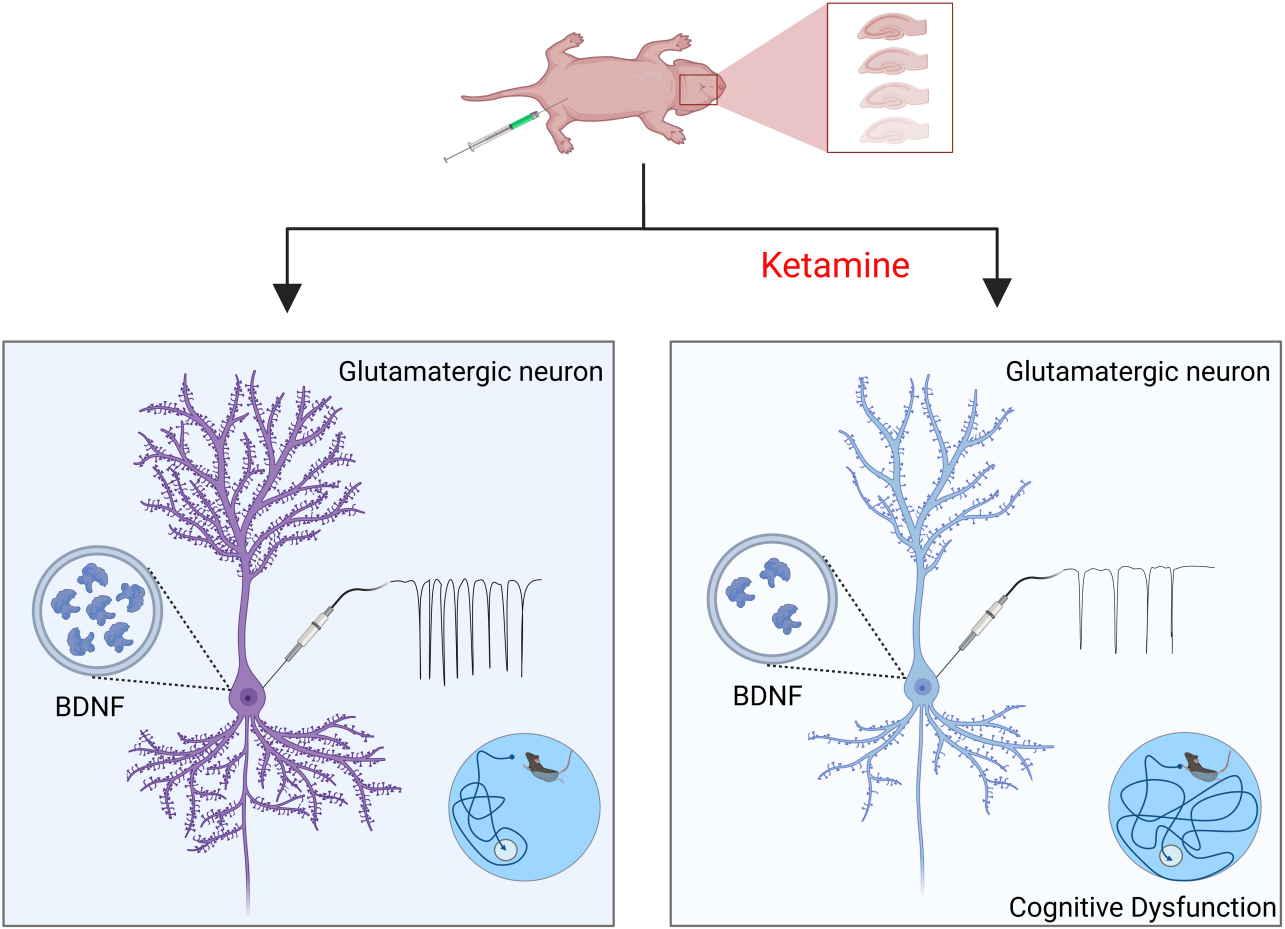
This figure illustrates the role of BDNF in cognitive dysfunction in adult mice that results from repeated ketamine exposure during development. After neonatal mice were anesthetized with ketamine multiple times, the level of BDNF in glutamatergic neurons in the CA1 area of the hippocampus was reduced, resulting in impaired neuronal synaptic plasticity and decreased neuronal excitability, ultimately leading to cognitive dysfunction in adulthood (created with BioRender.com).

In summary, our study shows that early exposure to ketamine for 3 consecutive days can lead to a reduction in BDNF levels in mouse hippocampal glutamatergic neurons, ultimately leading to the significant impairment of hippocampal synaptic plasticity and neuronal excitability and the development of cognitive dysfunction in adult mice (Figure 6). However, specifically increasing the expression level of BDNF in the brain and restoring the excitability of glutamatergic neurons can prevent and restore ketamine‐induced damage. These findings may contribute to a deeper understanding of the neurotoxicity caused by neonatal general anesthetic exposure and provide insights for the development of protective drugs.

## CONFLICT OF INTEREST STATEMENT

The authors declare no conflict of interests.

## Supporting information


**Figure S1.**.

**Data S1.**.

## Data Availability

The data that support the findings of this study are available from the corresponding author upon reasonable request.
