# Supplementary material for: Impaired synaptic plasticity and decreased excitability of hippocampal glutamatergic neurons mediated by BDNF downregulation contribute to cognitive dysfunction in mice induced by repeated neonatal exposure to ketamine
Source: CNS Neurosci Ther. 2024 Feb 8;30(2):e14604. doi: 10.1111/cns.14604 (PMC10853651; doi:10.1111/cns.14604)
Supplement: Supplementary file 1 — Figure S1.. [file CNS-30-e14604-s001.docx]

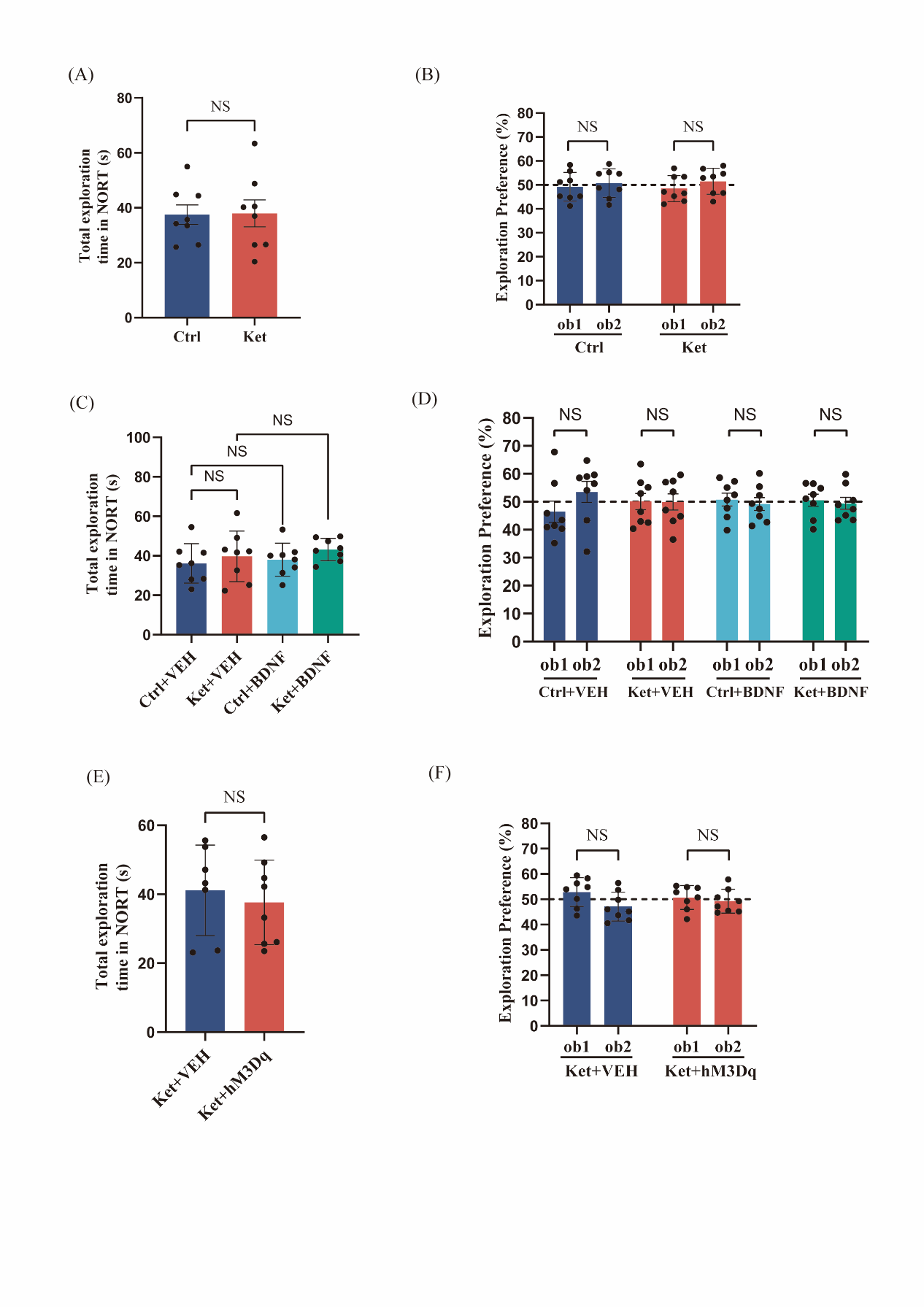


**Figure S1. Total exploration time and exploration preference in the training phase in NORT.** (A, C, and E) The total exploration time of objects shows no difference among different groups. (B, D, and F) The exploration preference for two objects in the training phase shows no difference among different groups. Statistical analyses were t-test. NS, no significance; ob1, object 1; ob2, object 2.
