# Supplementary material for: Impaired synaptic plasticity and decreased excitability of hippocampal glutamatergic neurons mediated by BDNF downregulation contribute to cognitive dysfunction in mice induced by repeated neonatal exposure to ketamine
Source: CNS Neurosci Ther. 2024 Feb 8;30(2):e14604. doi: 10.1111/cns.14604 (PMC10853651; doi:10.1111/cns.14604)

The lanes of the unedited blot that appear in the cropped image in the manuscript have been highlighted in red box.

Full unedited blot for Figure 1A

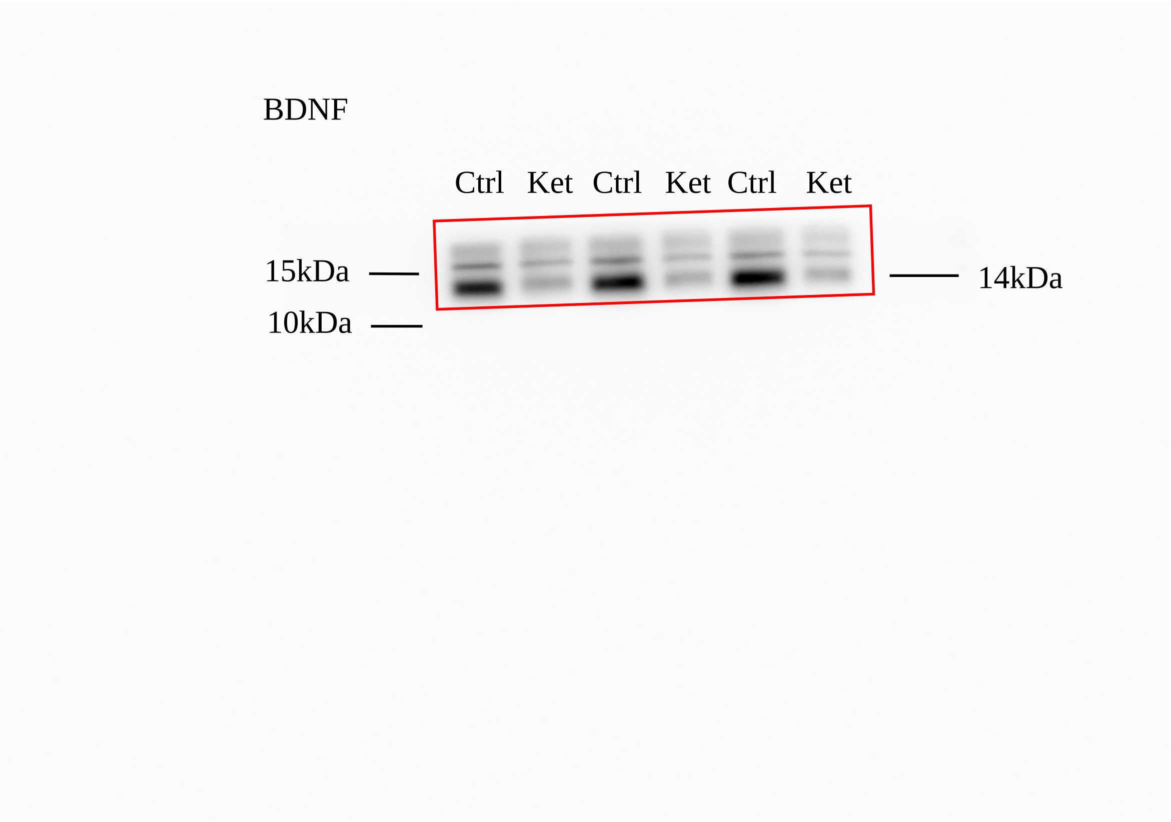

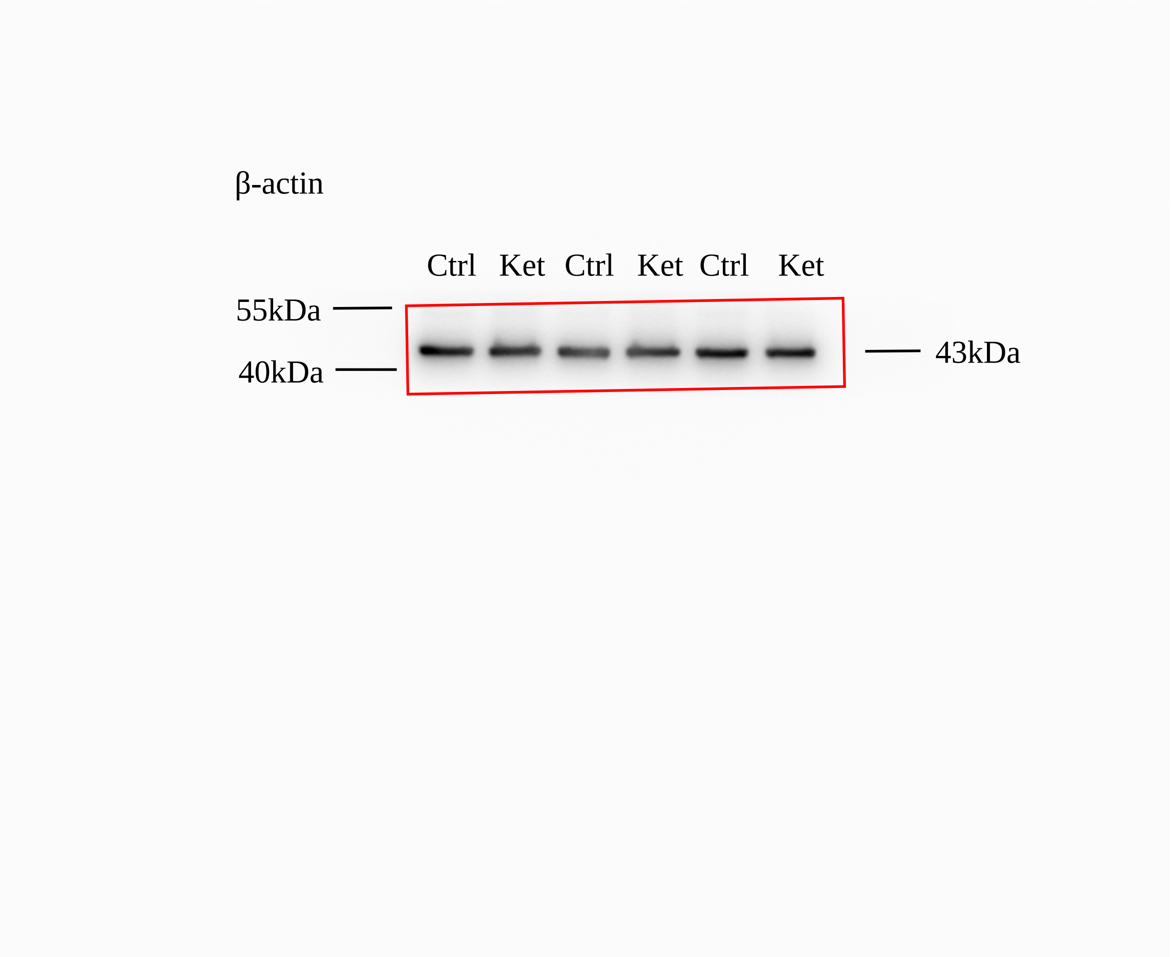

Full unedited blot for Figure 1G

PSD-95

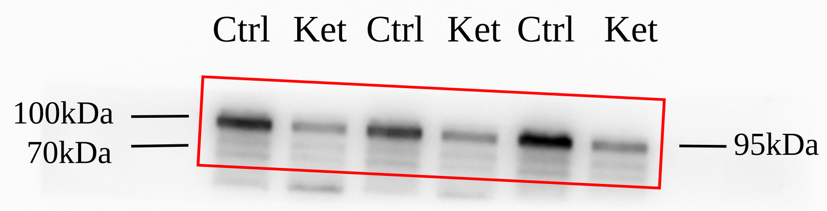

β-actin

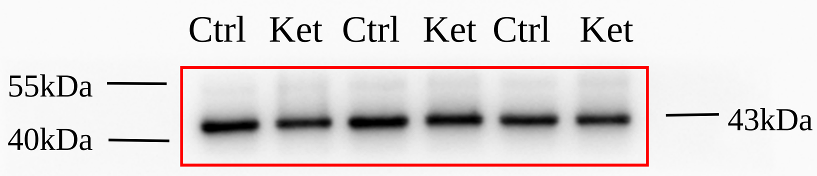

Full unedited blot for Figure 3B

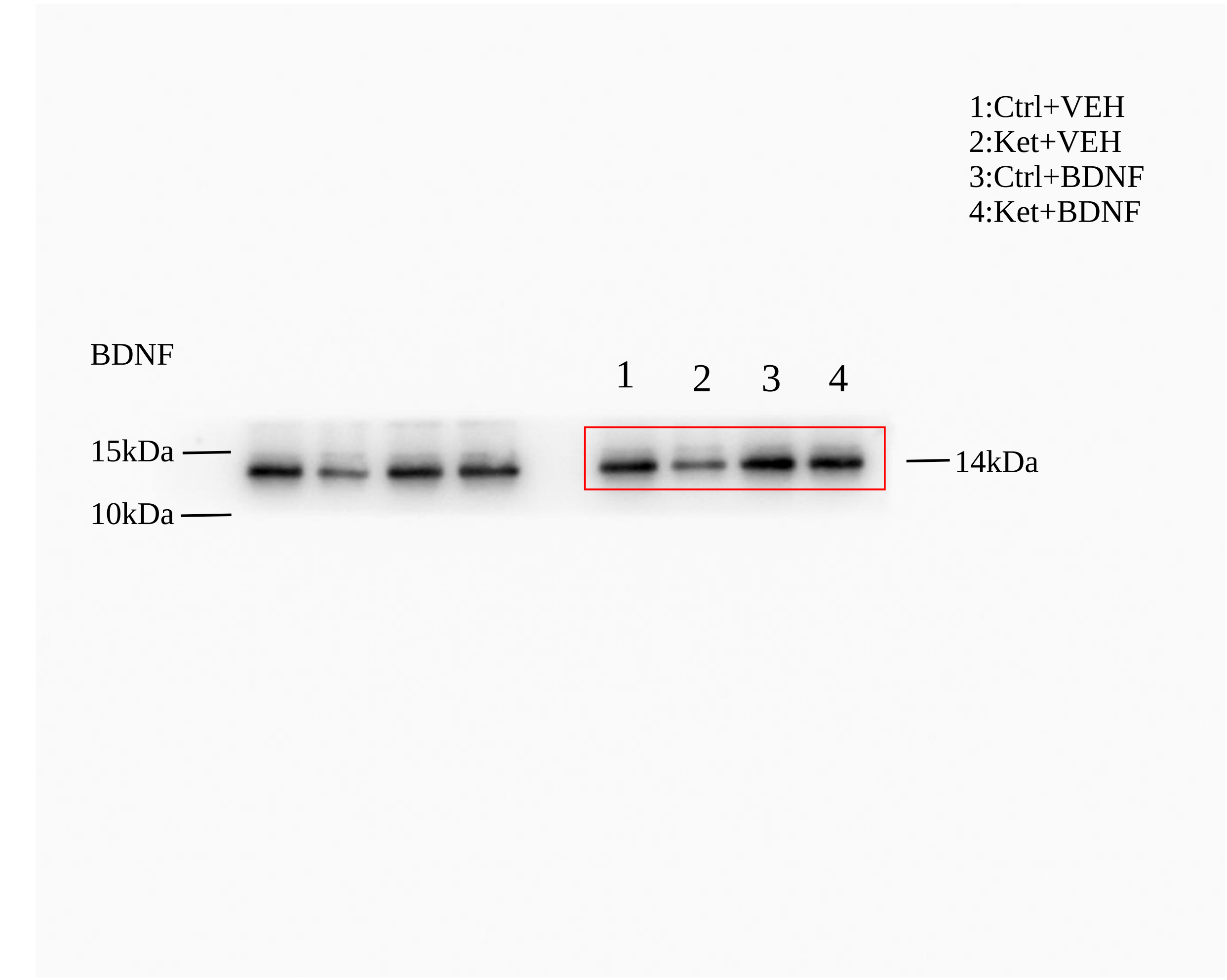

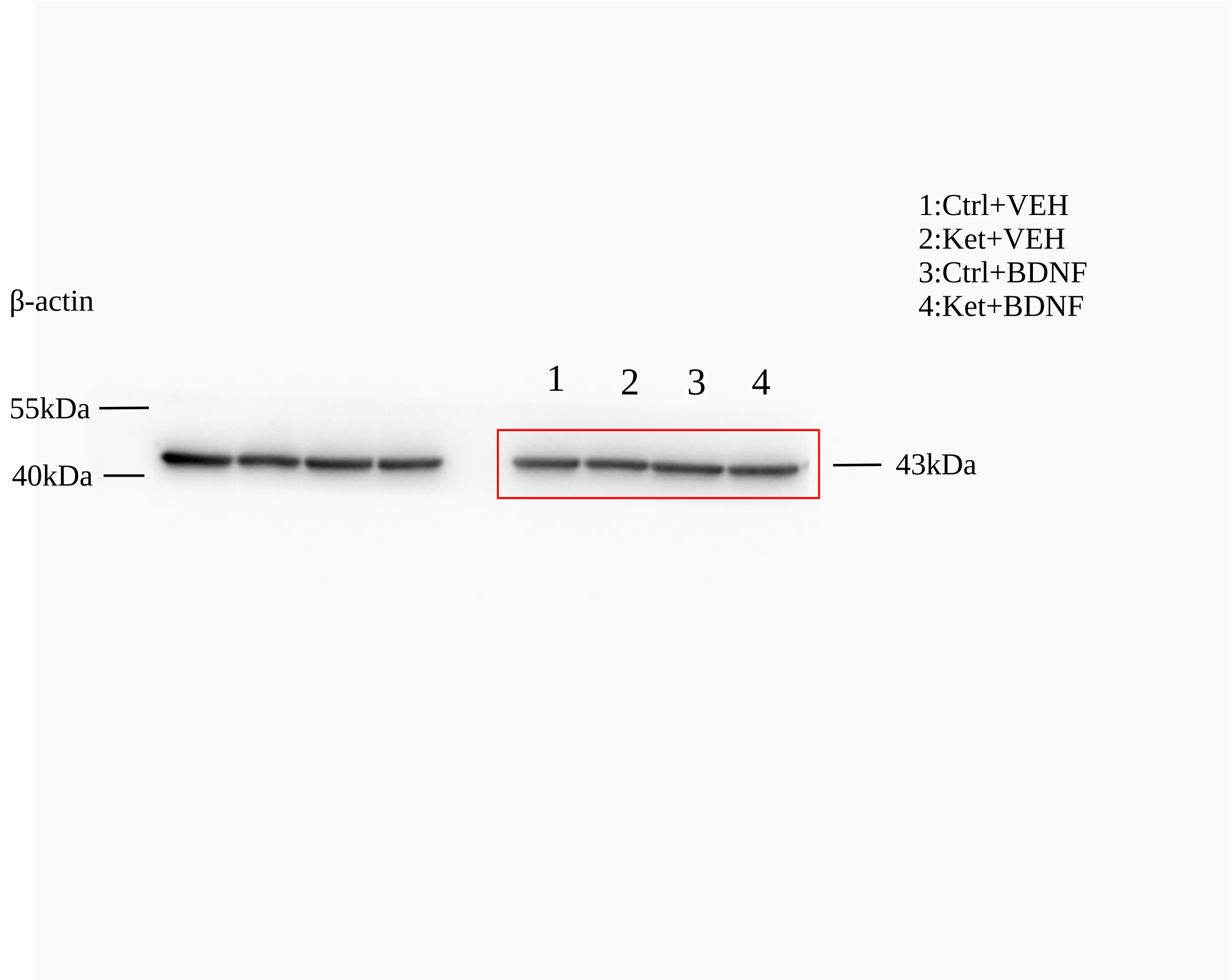

Full unedited blot for Figure 3F

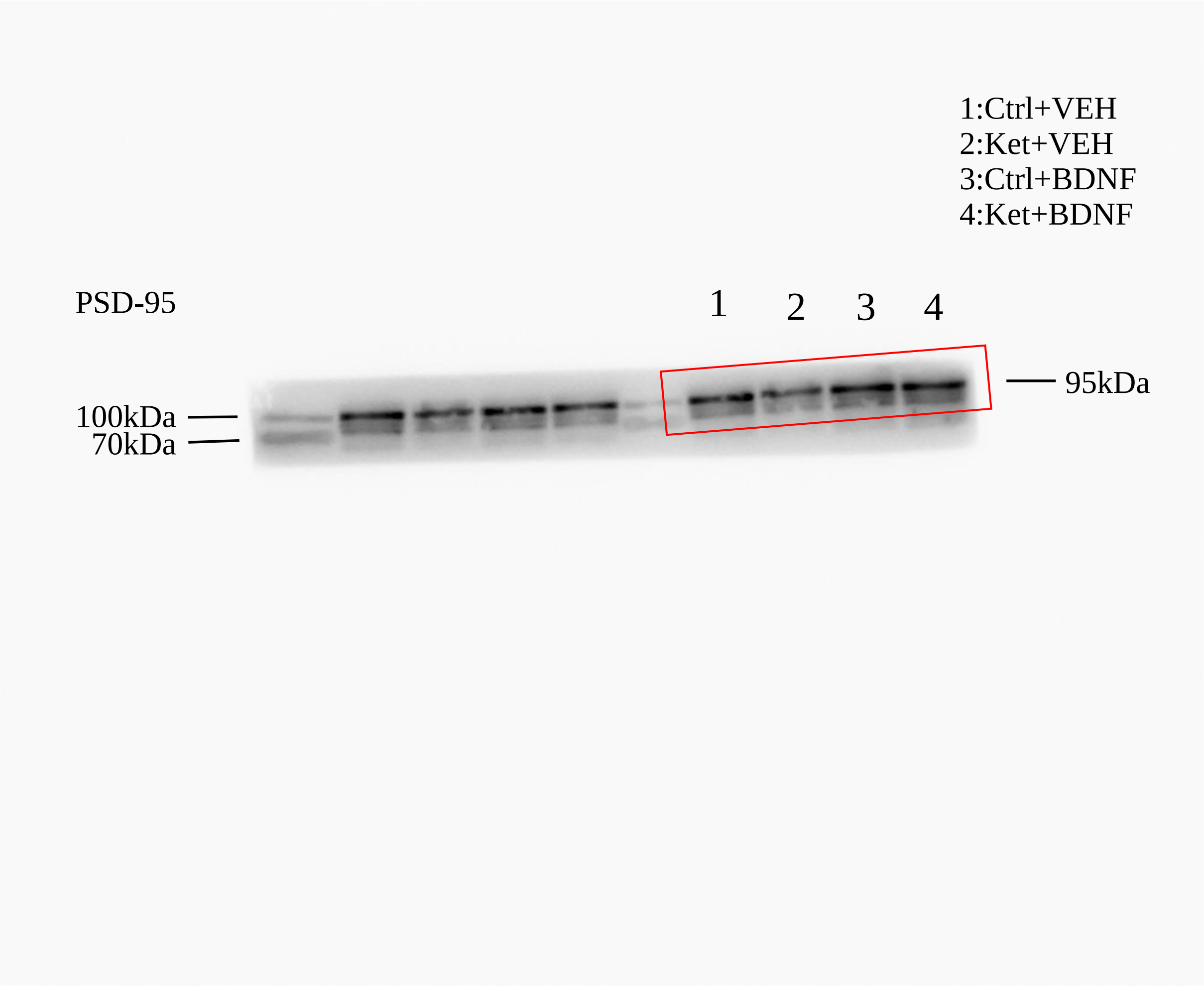

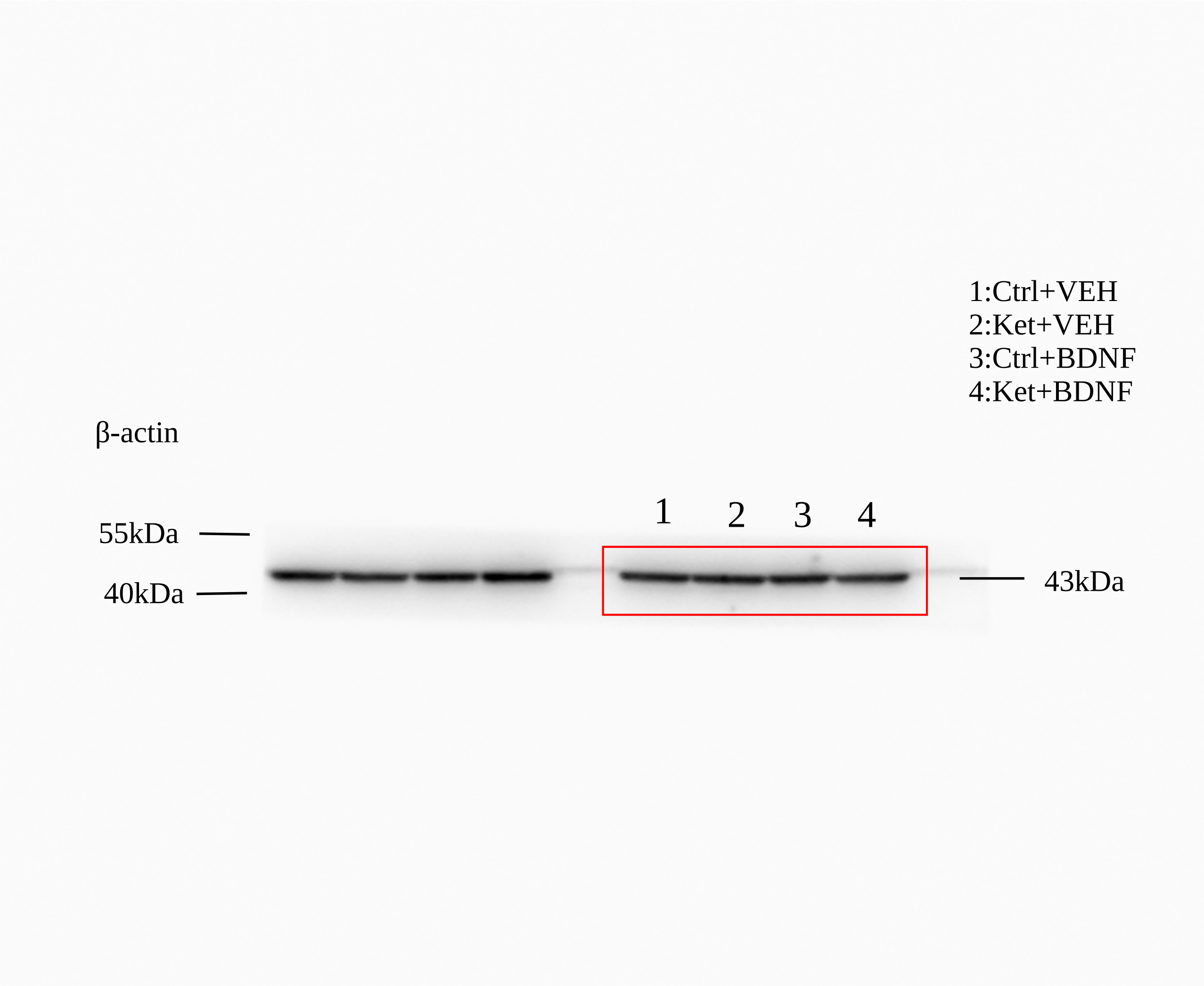

Supplement: Supplementary file 2 — Data S1.. [file CNS-30-e14604-s002.pdf]
